# Second primary malignancies in chronic lymphocytic leukaemia: Skin, solid organ, haematological and Richter's syndrome

**DOI:** 10.1002/jha2.366

**Published:** 2021-12-13

**Authors:** Yandong Shen, Luke Coyle, Ian Kerridge, William Stevenson, Christopher Arthur, Naomi McKinlay, Keith Fay, Christopher Ward, Matthew Greenwood, Oliver Giles Best, Ann Solterbeck, Alexander Guminski, Stephen Shumack, Stephen P. Mulligan

**Affiliations:** ^1^ Department of Haematology Royal North Shore Hospital St Leonards New South Wales Australia; ^2^ Kolling Institute of Medical Research Royal North Shore Hospital St Leonards New South Wales Australia; ^3^ College of Medicine and Public Health Flinders University Bedford Park South Australia Australia; ^4^ Statistical Revelations Pty Ltd Ocean Grove Victoria Australia; ^5^ Department of Medical Oncology Royal North Shore Hospital St Leonards New South Wales Australia; ^6^ Department of Dermatology Royal North Shore Hospital St Leonards New South Wales Australia

**Keywords:** chronic lymphocytic leukaemia, Richter's syndrome, second malignancies, skin cancer

## Abstract

Chronic lymphocytic leukaemia (CLL) is invariably accompanied by some degree of immune failure, and CLL patients have a high rate of second primary malignancy (SPM) compared to the general population. We comprehensively documented the incidence of all forms of SPM including skin cancer (SC), solid organ malignancy (SOM), second haematological malignancy (SHM) and separately Richter's syndrome (RS) across all therapy eras. Among the 517 CLL/small lymphocytic lymphoma (SLL) patients, the overall incidence of SPMs with competing risks was SC 31.07%, SOM 25.99%, SHM 5.19% and RS 7.55%. Of the 216 treated patients, 106 (49.1%) had at least one form of SPM, and 63 of 106 (29.2% of treated patients) developed an SPM 1.5 years (median) after treatment for their CLL. Melanoma accounted for 30.3% of SC. Squamous cell carcinoma (SCC), including eight metastatic SCCs, was 1.8 times more than basal cell carcinoma (BCC), a reversal of the typical BCC:SCC ratio. The most common SOMs were prostate (6.4%) and breast (4.5%). SHM included seven acute myeloid leukaemia (AML) and five myelodysplasia (MDS) of which eight (four AML, four MDS) were therapy‐related. Any SPM occurred in 32.1% of 53 Monoclonal B‐lymphocytosis (MBL) patients. Age‐adjusted standardised rates of SPM (per 100,000) for CLL, MBL and the general Australian population were 2648, 1855 and 486.9, respectively. SPMs are a major health burden with 44.9% of CLL patients with having at least one SPM, and apart from SC, associated with significantly reduced overall survival. Dramatic improvements in CLL treatment and survival have occurred with immunochemotherapy and targeted therapies, but mitigating SPM burden will be important to sustain further progress.

## 1 INTRODUCTION

Immune failure associated with chronic lymphocytic leukaemia (CLL) and small lymphocytic lymphoma (SLL) is one of the major and ongoing challenges of the disease. CLL immune failure results in an increased infection risk [1, 2, 3, 4] but also a significant rise in the incidence of second primary malignancies (SPMs). The advent of immunochemotherapy (ICT) and targeted therapies have significantly improved CLL‐related survival, but this enables the emergence of SPMs including skin cancer (SC) and solid organ malignancies (SOM), second haematological malignancies (SHM) such as myelodysplasia (MDS) and acute myeloid leukaemia (AML) and high‐grade lymphoid malignancies, that is, Richter's syndrome (RS).

The higher risk of SPM associated with CLL has been recognized for many years, well prior to the modern therapy era [5, 6, 7, 8, 9, 10]. Manosow and Weinerman [5] in 1975 demonstrated a three‐fold increase of SPM and an eight‐fold increase of SC in CLL. Data from M.D. Anderson Cancer Centre (MDACC) in 2009, after the introduction of ICT but prior to the targeted therapy era, showed a 2.2‐fold higher risk of SPMs in CLL/SLL compared to the general population [7]. In more recent reports, Ishdorj et al. [8] in 2019 demonstrated a four‐fold increase in SC with decade‐long follow‐up of the Manitoba CLL population, while Bond et al. [11] in 2020 showed a 2.2‐fold higher rate of SPM among CLL patients treated with BTK inhibitors.

As noted by others, SPM risk in CLL has not been systematically addressed [12]. In this detailed and comprehensive analysis from a single institution over 40 years, we evaluated all four major forms of SPMs assessed as separate entities viz; SC, SOM, SHM, and RS, that occur in CLL (including CLL, SLL and separately monoclonal B‐lymphocytosis [MBL]). Furthermore, we compared the incidence rates of SPM with the CLL literature and with Australian Cancer registry and Australian Institute of Health and Welfare (AIHW) which provides Australian population data for malignancy.

## 2 METHODS

### 2.1 Patient cohort

Data included in this study were obtained from patients managed at Royal North Shore Hospital (RNSH), Sydney, NSW, Australia, with informed consent. Research was approved by the Northern Sydney Local Health District Human Research Ethics Committee (approval number: LNR/14/HAWKE/181). RNSH is the principal hospital for Northern Sydney with a population of ∼1.3 million, the primary source of patients; in addition to which there are specialist referrals to this teaching hospital from across the state of New South Wales (population ∼8 million). All CLL, SLL and MBL patients in the Department of Haematology Audit 4 (S4S) data base were reviewed in detail including every histopathology report and all communications for every patient for the period January 1981 to December 2020. Patients were included in the analysis if there was over 1 year of follow‐up. Patient age was calculated at the last follow‐up.

CLL, SLL and MBL patients were diagnosed in accordance with international workshop on CLL guidelines [13]. CLL and SLL were analysed as one disease, hereafter referred to as 'CLL'. Follow‐up time was calculated from diagnosis of CLL to the last follow‐up date. MBL was analysed separately.

All SPMs, including those diagnosed prior to CLL, were summarised in the raw incidence. SCs, including metastatic SC, were assessed separately to SOMs. Diagnosis of SHMs was diagnosed based on morphology, histology, phenotype and genetics typing following standard criteria of the WHO classification [14]. The myeloid malignancies AML or MDS, prior CLL therapy exposed and not, were assessed separately to myeloproliferative neoplasms (MPN). Likewise, low‐grade lymphoid malignancies defined as hairy cell leukaemia (HCL), follicular lymphoma (FL), mantle cell lymphoma (MCL), monoclonal gammopathy of undetermined significance (MGUS) and multiple myeloma (MM), were assessed separately. RS followed the standard definition of a 'high‐grade lymphoid malignancy' in a patient with pre‐existing CLL. Mostly in the literature, and our cohort, these were diffuse large B‐cell lymphoma (DLBCL), with smaller numbers with Hodgkin's lymphoma (HL). Rarely, T‐cell variants have been described, and we included in the RS group T‐cell malignancies of any histology [15, 16].

### 2.2 Statistical analysis

Statistical analysis and cumulative incidence (and 95% confidence limits [CLs]) of SPM was determined using the method of fine and gray with death as a competing risk. All statistical analyses were performed using SAS software (V14.3). Overall survival (OS) was determined using the Kaplan–Meier method and comparisons between specific SPMs and the cohort with CLL only performed using the log‐rank test. Hazard ratios (HRs) were calculated using a Cox proportional hazards model with age group and gender as covariates.

To obtain annual average age‐standardised rates (ASR), the crude rate for each age group in 5‐year age bracket was determined as the number of individuals with malignancy of interest/total person years, multiplied by 100,000 (to express rate as number per 100,000). The crude rate was then multiplied by the proportion of individuals in that age group according to Australian Bureau of Statistics (ABS) data (2001 Australian standard population). The overall average annual age‐adjusted rate (per 100,000) was the sum of the adjusted rates for each age group. This was compared with data provided by the AIHW, an independent statutory Australian Government agency that provides health‐related information and statistics (aihw.gov.au) [17]. Standardised incidence ratio (SIR) was calculated as the ASR for our cohort/ASR from the AIHW dataset. CLs were calculated using the methods described by Rothman et al. [18].

## 3 RESULTS

### 3.1 Patient characteristics

A total of 570 patients diagnosed with CLL (501), SLL [16] or MBL (53) from January 1981 to December 2020 with a minimum 1‐year follow‐up were included. Median follow‐up duration was 11 years. The male to female (M:F; 323:194) ratio of the CLL cohort was 1.7:1 with a median age of 74.0 and age range of 18 to 101 years. The follow‐up time of CLL ranged from 1 to 39 years, during which 122 patients recorded an SC (23.6%), and 103 recorded an SOM (19.9%), respectively. There were 30 cases with an SHM, not including 31 RS discussed separately. Overall, 232 of 517 (44.9%) CLL patients had at least one SPM (Table 1) (including SPMs diagnosed prior to the CLL follow‐up period).

**TABLE 1:** Clinical features of chronic lymphocytic leukaemia (CLL) and monoclonal B‐lymphocytosis (MBL) patients

	Number of patients	Male : Female	Median age at the last follow‐up (inter‐quartile)	Mortality
**CLL cohort**	517	1.7 : 1	74.0 (66.0–83.0)	15.5%
CLL only	285 (55.1%)	1.5 : 1	73.0 (62.0–82.0)	11.9%
Skin cancer	122 (23.6%)	2.0 : 1	77.5 (70.3–84.0)	14.8%
Solid organ malignancies	103 (19.9%)	2.0 : 1	76.0 (69.0–84.0)	19.4%
Haematological malignancies	30 (5.8%)	2.3 : 1	75.0 (66.0–80.8)	43.3%
Richter's syndrome	31 (6.0%)	2.4 : 1	68.0 (63.3–73.0)	35.5%
Any SPMs*	232 (44.9%)	1.9 : 1	76.0 (68.8–84.0)	19.8%
**MBL cohort**	53	1.6 : 1	75.5 (67.0–82.0)	5.7%
MBL only	36 (67.9%)	1.8 : 1	75.5 (67.0–80.5)	5.6%
Skin cancer	9 (17.0%)	1.3 : 1	84.0 (75.0–92.0)	11.1%
Solid organ malignancies	7 (13.2%)	0.4 : 1	78.0 (73.0–81.5)	0.0%
Any SPMs*	17 (32.1%)	1.8 : 1	78.0 (67.3–84.8)	5.9%

Abbreviation: SPM, second primary malignancy.

* Patients with at least one or more diagnosis of either skin, solid organ malignancies, second haematological malignancies or Richter's syndrome, in addition to their CLL or MBL. Patients diagnosed with multiple types of SPMs were counted as separate incidences. Median age was calculated at the last follow‐up or the age when patient died.

Of the 517 CLL patients, immunoglobulin heavy chain variable region genes (IGHV) status was available on 38 (7.4%, 27 mutated and 11 unmutated) and cytogenetics analysis on 106 patients (20.5%). Of the 11 unmutated patients, there were five SCs, four SOMs, one myeloma and no RS recorded; of the 27 mutated patients, there were six SCs, four SOMs and no SHM or RS.

A total of 216 patients had at least one line of treatment. Of first‐line treatments, 66 were alkylating/chlorambucil based, 125 fludarabine, cyclophosphamide, rituximab (FCR, or other fludarabine) based, 10 targeted therapies and 15 others not specified (Figure S1). Most targeted therapy (68 patients) was for relapsed CLL. Of the 216 treated patients, 106 (49.1%) had at least one form of SPM, and 63 of 106 (29.2% of treated patients) developed an SPM 1.5 years (median) after treatment for their CLL; 24 had an SPM before CLL treatment; and in 19 the sequence was unclear.

There were 53 patients with MBL (Table 1) with a median age of 75.5 years and M:F ratio of 1.6:1 (33:20). Of the 53 MBL patients, nine (17.0%) had SC (five melanoma, seven non‐melanoma SCs [NMSCs], including two basal cell carcinoma [BCC], three squamous cell carcinomas (SCCs), one sebaceous cell carcinoma and one unclassified); and seven (13.2%) had SOMs, two with prostate cancer and one each of breast, bladder, thyroid (papillary cell), pancreas and colon. There were two MBL patients with DLBCL consistent with RS and two diagnoses of MBL with MGUS.

### 3.2 Second malignancy in the CLL cohort

#### 3.2.1 SC

Of the 517 CLL patients, 122 had an SC (23.6%), including those diagnosed prior to CLL (Table 2). Among these 122 patients, 18 had SC prior to the diagnosis of CLL with a median age 69.0 years, 59 were diagnosed after the CLL with median age of 62.0 years, and 30 patients could not be chronologically traced (Figure S2). Melanoma accounted for 37 (30.3% SCs; M:F ratio 1.5:1) of the 122 SC patients, while there were 101 (82.8% SCs; M:F ratio 2.4:1) NMSCs (BCC and SCC) (16 had both melanoma and NMSC, with 14 NMSCs not classified). We observed 33 patients with BCC (M:F ratio 0.9:1) and 58 with SCC (M:F ratio 3.5:1). Metastatic SCC occurred in eight (13.8% SCC; 6.6% SCs). Two patients had Merkel cell carcinoma (2.0% NMSC; 1.6% SCs), and three had CLL skin infiltration; the latter are included to indicate relative incidence but are not included in the 'SC' analysis (Table 2).

**TABLE 2:** Skin cancers and solid organ malignancies in the chronic lymphocytic leukaemia (CLL) cohort

			Male		Female	
**Skin cancers**	Cases	% of CLL cohort	Cases	% in male (81)	Cases	% in female (41)
Melanoma	37	7.2%	22	27.2%	15	36.6%
NMSC	102	19.8%	72	88.9%	30	73.2%
BCC	33	6.4%	16	19.8%	17	41.5%
SCC	58	11.2%	45	55.6%	13	31.7%
Merkel	2	0.4%	1	1.2%	1	2.4%
Sebaceous cell carcinoma*	1	0.2%	0	0.0%	1	2.4%
CLL skin infiltration**	3	0.6%	3	3.7%	0	0.0%
**Solid organ malignancies**	Cases	% of CLL cohort	Cases	% in male (70)	Cases	% in female (33)
Prostate	33	6.4%	33	47.1%	0	0.0%
Breast	23	4.5%	0	0.0%	23	69.7%
Lung	12	2.3%	8	11.4%	4	12.1%
Colorectal	11	2.1%	8	11.4%	3	9.1%
Kidney	5	1.0%	4	5.7%	1	3.0%
Endocrine (pituitary, thyroid, parathyroid)	5	1.0%	3	4.3%	2	6.1%
Bladder	4	0.8%	4	5.7%	0	0.0%
Pancreas	3	0.6%	3	4.3%	0	0.0%
Esophagus	3	0.6%	3	4.3%	0	0.0%
Schwannoma	3	0.6%	3	4.3%	0	0.0%
Brain (glioma, meningioma)	2	0.4%	1	1.4%	1	3.0%
Testicular	2	0.4%	2	2.9%	0	0.0%
Ovarian	1	0.2%	0	0.0%	1	3.0%
Endometrial	1	0.2%	0	0.0%	1	3.0%
Chondrosarcoma	1	0.2%	1	1.4%	0	0.0%
Lymph node metastasis (UPC***)	1	0.2%	1	1.4%	0	0.0%

*Note*: Numbers in this table indicate all CLL patients with skin cancers or solid organ malignancies including those diagnosed prior to their CLL. NMSC incidences included BCC, SCC, Merkel cell carcinoma and cases of unclassified NMSC cases. Patients diagnosed with multiple types of skin cancers were counted as separate incidences.

Abbreviations: BCC, basal cell carcinoma; NMSC, non‐melanomatous skin cancer; SCC, squamous cell carcinoma.

* Sebaceous cell carcinoma was identified in an MBL patient and is not included in the CLL analysis cohort.

** CLL infiltration not included in 'skin cancer' statistics and provided only for comparison.

*** UPC, uncertain primary carcinoma.

A total of 57 patients with SC were treated for their CLL, 26 (21.3% SCs) before the diagnosis of SC (four chlorambucil based, 11 fludarabine based, eight targeted therapies and three others); 12 had no CLL treatment prior to SC diagnosis (9.9% SCs); and 19 could not be chronologically traced. The median time of between treatment of CLL and diagnosis of SC was 18 months.

#### 3.2.2 SOMs

There were 103 patients (19.9%) diagnosed with one or more SOMs including those diagnosed prior to their CLL (Table 2). Prostate and breast cancers were the most common SOMs in the CLL cohort, 33 prostate cancers (47.1% male SOMs) and 23 breast cancers (69.7% female SOMs), respectively. Lung and colorectal cancers ranked third and fourth. Details of other SOMs and the diagnosis time relative to the CLL are shown in Table 2 and Figure S3.

Of the 103 patients, 19 (18.4% SOMs) were treated for their CLL (four chlorambucil based, eight fludarabine based, five targeted therapies and two others) prior to the diagnosis of SOMs. Median time between those treatments and diagnosis of SOMs was 12 months.

#### 3.2.3 Secondary haematological malignancies

##### Myeloid neoplasms

AML and MDS were diagnosed in seven and five patients, respectively (Table 3). Of the seven AML cases, four were previously treated, two with FCR, and of the five MDS cases, four were CLL treated, three with FCR, totalling 8/12 'therapy‐related' (66.7%) AML/MDS (t‐AML/MDS). Of the 124 CLL patients treated with FCR (not including other fludarabine‐based therapy) in any line of treatment, five developed t‐AML/MDS (4.0%, [or 3.1% with all fludarabine‐based therapy included]. Treatment history of the 12 AML/MDS patients is summarised in Table S1.

**TABLE 3:** Second haematological malignancies in the chronic lymphocytic leukaemia (CLL) cohort

				Male (21)		Female (9)	
	Cases	% of CLL cohort	Prior Rx for CLL	Cases	% in male	Cases	% in female
AML and MDS	12		8				
9AML	7	1.4%	4	7	23.3%	0	0.0%
MDS	5	1.0%	4	3	10.0%	2	20.0%
Other MPNs	6		1				
Myelofibrosis	2	0.4%	1	1	3.3%	1	10.0%
Essential thrombocythemia	2	0.4%	0	1	3.3%	1	10.0%
Polycythemia vera	1	0.2%	0	1	3.3%	0	0.0%
CML	1	0.2%	0	0	0.0%	1	10.0%
LPD	7		1				
ALL*	1	0.2%	1	0	0.0%	1	10.0%
LPD (inc. FL, HCL and MCL)	6	1.2%	1	2	8.0%	4	40.0%
Immunoproliferative disorders	6		0				
MGUS	4	0.8%	0	4	13.3%	0	0.0%
Multiple myeloma	2	0.4%	0	1	3.3%	1	10.0%

*Note*: Numbers in this table indicate all CLL patients with second haematological malignancies including those diagnosed prior to their CLL.

Abbreviations: AML, acute myeloblastic leukaemia; CML, chronic myeloid leukaemia; FL, follicular lymphoma; HCL, hairy cell leukaemia; LPD, low‐grade lymphoproliferative disorder; MCL, mantle cell lymphoma; MDS, myelodysplastic syndromes; MGUS, monoclonal gammopathy of undetermined significance; MPN, myeloproliferative neoplasm.

* ALL, acute lymphoblastic leukaemia. One case of ALL included in the Richter's syndrome (RS) analysis, not counted as 'LPD' but included for comparison with other second haematological malignancy (SHM). See Supporting data regarding the ALL patient.

All 12 cases of AML or MDS were diagnosed after the CLL at a median time of 6 and 10 years, respectively. The 'therapy‐related' four AML and four MDS occurred at median time from CLL therapy of 3 and 3 years, respectively. The median survival (calculated from the time of AML or MDS diagnosis) after AML or MDS diagnosis was 4 and 24 months, respectively. To date, two patients have developed AML after targeted therapy, although follow‐up is much shorter (median 36 months), and both had received prior ICT. The three AML cases with no prior CLL therapy occurred 4, 7 and 8 years after the diagnosis of CLL; survival after AML diagnosis was 1, 1 and 4 months at ages 60, 70 and 80 years. The one MDS case with no prior CLL therapy (aged 87) was alive at study cut‐off date, 36 months after MDS diagnosis.

MPN was diagnosed in six patients (1.2%) including two idiopathic myelofibrosis (MF), two essential thrombocythemia (ET), one polycythaemia vera (PV), and one chronic myeloid leukaemia (CML) (Table 3). Of these MPNs, two MF, one PV and one CML were diagnosed after the CLL, and one MF patient was previously CLL treated with FCR 1 month earlier. The two cases of ET were diagnosed prior to CLL with no prior treatment for the ET.

##### Other lymphoproliferative and plasma cell disorders

There were six patients (two FL, three MCL and one HCL) diagnosed with a second lymphoproliferative disorder (Table 3). Both FLs were diagnosed before the CLL, including an unusual concurrently diagnosed combination of three haematological malignancies: SLL and FL in a lymph node biopsy (documented distinct by histology and immunohistochemistry) and DLBCL (hence RS) in a gastric biopsy. Diagnosis of CLL and HCL was made concurrently in a 64‐year‐old female from blood morphology and distinctive immunophenotypes. There were three CLL patients with confirmed MCL by phenotype and genetics, all males aged 45, 51 and 61 years at CLL diagnosis. One (age 45) was diagnosed concurrently and the other two after the CLL. The male aged 51 was then diagnosed with MCL at age 55 years, and then AML 5 years later aged 60 and died 1 month later (hence included in myeloid neoplasm analysis).

There were six CLL patients with plasma cell dyscrasias, four MGUS and two MM. Of these, four (three MGUS and one MM) were diagnosed within 6 months after the CLL. The other two were diagnosed with MGUS or MM 13 years after CLL. One patient with MGUS and one MM were treated with obinutuzumab plus chlorambucil and FCR, respectively, for their CLL, after diagnosis of MGUS/MM.

##### RS

RS occurred in 31 patients including 22 DLBCL (73.3% of RS) and four HL (13.3% of RS). We included all high‐grade lymphoid malignancies, both of B‐cell type (two B‐cell prolymphocytic leukaemia), one B‐ALL and two T‐cell malignancies (one aggressive peripheral T‐cell lymphoma‐not otherwise specified and one subcutaneous panniculitis‐like T‐cell lymphoma) (Table 4). The one ALL patient was not on lenalidomide (See Table S2). RS was diagnosed at a median 6 years after the CLL and 1.5 years (median) after the last CLL treatment. The median survival (from onset of RS) after RS diagnosis was 24 months.

**TABLE 4:** Richter's syndrome

				Male (22)		Female (9)	
	Cases	% of CLL cohort	Duration from CLL Dx*	Cases	% in male	Cases	% in female
DLBCL	22	73.3%	6 years	17	81.0%	5	55.6%
Hodgkin's lymphoma	4	13.3%	9 years	2	9.5%	2	22.2%
B‐cell prolymphocytic leukemia	2	6.7%	4.5 years	2	9.5%	0	0.0%
T‐cell lymphoma	2	6.7%	6.5 years	1	4.8%	1	11.1%
B‐ALL*	1	3.3%	11 years	0	0.0%	1	11.1%

*Note*: Table includes two MBL patients with Richter's syndrome (RS). Numbers in this table indicate all CLL patients with Richter's syndrome including those diagnosed prior to their CLL.

Abbreviations: ALL, acute lymphoblastic leukaemia; CLL, chronic lymphocytic leukaemia; DLBCL, diffuse large B‐cell lymphoma.

* Duration from CLL diagnosis (Dx) indicates the median time between diagnosis of CLL and RS.

### 3.3 OS and competing risk analysis

**FIGURE 1:**
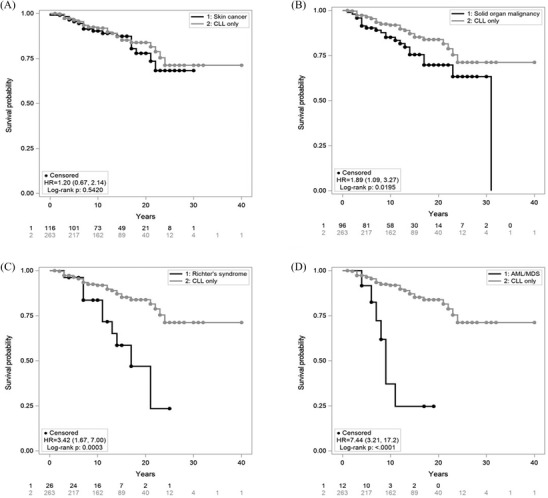
Overall survival (OS) of chronic lymphocytic leukaemia (CLL) patients with a second primary malignancy. The survival time was calculated from the time of the CLL diagnosis. (A) analysis by skin cancer; (B) analysis by solid organ malignancies; (C) analysis by Richter's syndrome; (D) analysis of acute myeloid leukaemia (AML) and myelodysplasia (MDS). *p*‐values were calculated using the Log‐Rank tests. *p*‐values <0.05 were considered statistically significant. Numbers at risk were listed for each panel under the x‐axis

The OS of CLL patients with and without any SPM was illustrated by the Kaplan–Meier curves (Figure 1). OS was worse with SOM (HR 1.89, *p = *0.0195), RS (HR 3.42, *p = *0.0003) and AML/MDS (HR 7.44, *p* < 0.001), but not for SC (HR 1.2, *p = *0.5420).

The occurrence of death before SPM can bias analyses of incidence. Therefore, calculation of overall incidence of SPMs in the CLL cohort was adjusted using death as a competing risk (Table 5). This analysis only considered incidences of SPMs developed after the diagnosis of CLL and excluded patients with missing key clinical information. The adjusted overall incidence of all SPM was 59.36%, SC was 31.07%, SOM was 25.99%, RS was 7.55%, SHM was 5.19% and AML/MDS was 2.71%. Cumulative incidence rates of SPMs by age groups at CLL diagnosis were higher in younger age groups for all types of SPMs except for SHM (Figure S4), that is, a higher risk of SPM during their longer‐time course of CLL.

**TABLE 5:** Summary of second primary malignancy (SPM) with death as a competing risk

Type of malignancy	Number developing SPM	Number dying before SPM	Number alive and disease free	Total number in analysis	Overall incidence (95% confidence limits)
Any malignancy	144	31	232	407	59.36% (47.36%, 69.49%)
Skin cancer	70	59	305	434	31.07% (22.76%, 39.72%)
Solid organ malignancy	47	55	329	431	25.99% (13.71%, 40.10%)
Richter's syndrome	24	67	386	477	7.55% (4.76%, 11.18%)
Haematological malignancy	19	66	385	470	5.19% (3.16%, 7.94%)
AML/MDS	9	70	398	477	2.71% (1.31%, 4.98%)

*Note*: Table summarises the overall incidence of SPMs in the chronic lymphocytic leukaemia (CLL) cohort using death as a competing risk. This analysis included SPMs diagnosed after the diagnosis of CLL only and excluded patients with incomplete information, hence discrepancies between raw incidence numbers and total numbers in the analysis.

Abbreviations: AML, acute myeloid leukaemia; MDS, myelodysplasia.

Analysis was performed to evaluate potential causal relationship between CLL therapy and SPM development. There was no treatment effect for SC (HR 1.16, CL 0.72, 1.87) or SOM (HR 0.95, CL 0.52, 1.71). RS (HR 2.18, CL 0.95, 5.01) did appear to have a treatment effect with 'any prior CLL therapy' and a marked effect with fludarabine therapy (HR 7.42, CL 0.92, 60.11). There appeared to be a treatment effect for AML/MDS (HR 3.15, CL 0.66, 15.01), but when fludarabine was compared to 'any other form of CLL therapy', this effect was lost (Figure S5). Both the RS and AML/MDS numbers were small, and these effects need to be examined on larger numbers of patients.

### 3.4 Age standard incidence of SPM for CLL compared to the Australian population

The age standard incidence (ASR) for the CLL and MBL cohort was calculated as described in Methods. The RNSH North Sydney Area closely matches the general Australian population (Figure S6). The ASR of SPMs for CLL and MBL (Table 6) were all substantially higher than the Australian population. AML and MDS had relatively small numbers resulting in wide confidence intervals.

**TABLE 6:** Summary of age‐adjusted rates using data for 2015 from Australian Institute of Health and Welfare (AIHW) report cancer in 2019, age adjustment using 2001 population data and comparison with the chronic lymphocytic leukaemia (CLL) and monoclonal B‐lymphocytosis (MBL) cohort

Type of malignancy	Age standardized rate for the general Australian population (per 100,000)	Age standardized rate for the CLL cohort (per 100,000, age in 5‐year brackets)	Standardized incidence ratio (95% confidence limits, age in 5‐year brackets)
Any malignancy	486.9	2648	5.44 (4.94, 5.99)
Melanoma	51.8	278.7	5.38 (4.00, 7.24)
Breast	64.7	320.2	4.95 (3.79, 6.46)
Prostate	140.9	332.7	2.36 (1.94, 2.88)
Lung cancer	42.8	94.4	2.21 (1.54, 3.16)
Colorectal cancer	57.4	184.8	3.22 (2.39, 4.33)
AML	3.9	1524	391 ( 145, 1056)
MDS	4.7	262.9	55.9 (22.5, 139)
Type of malignancy	Age‐standardized rate for the general Australian population (per 100,000)	Age‐standardized rate for the MBL cohort (per 100,000, age in 5‐year brackets)	Standardized incidence ratio (95% confidence limits, age in 5‐year brackets)
Any malignancy	486.9	1855	3.81 (3.45, 4.21)

*Note*: Age standard rates (ASR) for the CLL cohort was calculated using methods described in the statistical analysis section. ASR for the general Australian population was obtained from the Australian Institute of Health and Welfare (AIHW) caner in Australian 2019 report. ASR was calculated for both 10‐ and 5‐year brackets with minimal differences; age in 5‐year brackets was shown.

Abbreviations: AML, acute myeloid leukaemia; MDS, myelodysplasia.

## 4 DISCUSSION

The development of an SPM has a strong association with CLL. This association long pre‐dates the advent of ICT and targeted therapy but appears to be a more significant clinical issue in the current era as patients survive much longer with their CLL. Following the introduction of ibrutinib in Australia in 2012 with the RESONATE trial [19], our centre observed, for the first time, higher mortality due to SC instead of CLL [20].

In the context of SC, melanoma is mandated as a cancer registry reportable malignancy in Australia, while NMSCs (BCCs and SCCs) numbers are only reflected by surveys and insurance records. SC in a 2009 audit at our institution and an NSW coastal centre was extremely common with NMSC in 58.9% (96/163) with differences between metropolitan versus regional centres (36.7% vs. 62.8%), likely reflecting lifestyle, occupation and different UV radiation. The incidence of melanoma was identical (9.8%). In this study, 23.6% of CLL patients had SC, with melanoma in 37 (7.2% of CLLs) and NMSC in 102 (19.8% of CLLs). Hence, the incidence of melanoma is comparable between 2009 and 2020 (9.8% vs. 7.2%, respectively), while the incidence of NMSC is lower (36.7% vs. 19.8%, respectively). This period corresponds with long‐term results from sun‐exposure education campaigns and suggests that NMSC risk in CLL can be modified. Comparison with a cohort of CLL patients from Manitoba, Canada, undergoing regular dermatological evaluation, showed comparable competing risk‐adjusted incidence of SC (31.07% vs. 28.0%) [8].

NMSC is not a reportable malignancy in Australia and indeed nor in many cancer registries. Staples et al. [21] showed the number of SCC in the Australian population was two‐times lower than BCC. By contrast, the incidence rate of SCC in our CLL cohort was 1.8‐times higher than BCC. This inversion of the SCC:BCC incidence rates in CLL is highly consistent with solid organ transplant patients on prolonged immunosuppression [22] and emphasizes immune failure contributing to NMSC risk. Furthermore, metastatic SCC (met‐SCC) occurred in eight patients (of 58 SCC; 13.8%). Comparison with the New Zealand (NZ) general community (with similar UV levels) reported 1.9%–2.6% met‐SCC [23], while an NZ CLL cohort [24] showed 9.9% (6/61) met‐SCC. Velez et al. [25] at two Boston academic centres had 10.7% (3/28) met‐SCCs over 20 years, demonstrating highly comparable met‐SCC rates in CLL in Australia, NZ and the USA, again supporting a role of immune failure.

Melanoma was the fourth most common cancer in Australia in 2019 with an ASR of 51.8 [26] but substantially higher in our CLL cohort (ASR 278.7, SIR 5.38, CL 4.0, 7.2, Table 6). Australian melanoma incidence rises with age with ASR from 80.2 for age 50–59 years to 229.1 over 80 years [27]. Similarly in CLL, both melanoma and NMSC rates rise with age with both substantially higher than general population highlighting both age [21] and CLL‐related immune failure as contributing factors for SC in CLL. In younger patients, we observed a higher relative incidence of SPM again consistent with immune failure (Figure S4).

SOMs were observed in 103 of the 517 CLL cohort. Consistent with the literature [12], prostate and breast cancer were the two largest contributors of SOMs; 47.1% of male cancers were prostate, and 69.7% of female cancers were breast cancers. AIHW in 2019 showed a community ASR of 140.9 for prostate and 64.7 for breast cancer. In our CLL cohort, the ASR was 2.46‐times higher (CL 2.02, 2.99) for prostate (346.4) and 4.66‐times higher (CL 3.56, 6.10) for breast cancer (320.2) (Table 6). Patients with SOMs had significantly higher mortality than patients with CLL only (*p *= 0.0195) with over 1.89‐fold higher risk of death (Figure 1), consistent with the literature [28]. The MBL cohort also showed comparable incidence rates for prostate and breast cancer. In CLL, lung and colorectal cancers were likewise 2.79‐times (CL 1.97, 3.96) higher (ASR 119.6 vs. 42.8) and 3.94‐times (CL 2.95, 5.26) higher (ASR 225.9 vs. 57.4) than the Australian population, respectively [26].

Focusing on AML and MDS, the 2.71% competing risk‐adjusted incidence (12/517) was higher in our CLL cohort compared to 0.6% by Lenartova et al. [29] in the Norwegian CLL registry. Australian community risk of AML to age 75 is 1 in 390 (0.26%) [30]. In our CLL cohort, AML total numbers, therapy‐related AML and de novo AML at 7 (1.4%), 4 (0.8%) and 3 (0.6%), respectively, are higher than population (Table 6 ASR AML in CLL 1524 vs. 3.9 population, SIR 391, CL 145, 1056). Our incidence of t‐AML/MDS at 4.0% (5/124) was very similar to the MDACC data [7] (5.1%; 12/234) calculated in the same manner. In our cohort, three of seven AML occurred with no prior CLL therapy. The Danish CLL registry of 4286 patients recorded 40 (0.93%) acute leukaemia, and 34 (85%) had no prior therapy [10]. They found a fludarabine risk for MDS but not acute leukaemia (only two of six treated received fludarabine) [10]. Our data suggest an 'any CLL therapy' effect but not a specific fludarabine effect (Figure S5), again with the important caveat of very low numbers. Our data and the Danish registry suggest that CLL itself represents a risk for AML (RNSH 0.6% untreated CLL vs. 0.26% Australian population; Danish 0.79% untreated CLL vs. 0.15% in ‘214,150 comparators’). Given how frequently AML risk is invoked as a reason to avoid FCR, this is an important issue to address in large CLL cohorts with treated and untreated patients. The coexistence of CLL and MPN in our cohort (1.2%) is similar to Italian (∼1%) [31] but higher than Danish data (0.16%).

RS occurred in 31 patients (7.55% adjusted incidence) (including two DLBCL within the 53 MBL), consistent with the incidence of RS in recent publications [32, 33, 34]. Over 70% of cases were DLBCL, with smaller numbers of HL and T‐NHL. Over half (17/30) had received treatment for their CLL, and 16 of those 17 were with FCR. Rossi et al. [35] and Parikh et al. [34] also reported over half of their RS patients were CLL treated. Statistics from MDACC showed the risk of RS following FCR was 6.6% [36], while the German CLL8 trial showed the risk was higher in the FC (6.3%) than the FCR arm (3.2%) [32]. Analysis using death as competing risk, the HR of RS in treated CLL versus untreated was 2.18 (CL 0.95, 5.01, Figure S5). There is insufficient evidence, due to small sample sizes and wide confidence intervals to determine whether RS is associated with fludarabine. Future investigation with larger sample sizes is warranted.

The comprehensive analysis of all forms of SPMs over a very long follow‐up is strength of this study. It accurately reflects the natural history of CLL in a large, mainly community‐based, single institution cohort with relatively uniform management. Very long follow‐up has potential ascertainment bias by selecting patients with more indolent CLL, and hence longer survival, perhaps enabling more of these patients to develop SPMs. CLL prognostic markers such as chromosomal mutations and IGHV status were available in a small proportion having become available late during this 40‐year long timeframe; in any event, staging and karyotype are dynamic factors that change with time. CLL patients when diagnosed with an SOM were usually referred to an oncologist occasionally limiting our ability to precisely identify the cause of death, but most subsequent mortality appeared due to the SOM rather than CLL.

The high incidence of SPMs (ASR 2648 in CLL vs. 486.9 in population) has a substantial impact and health burden upon CLL patients, their management and survival. Routine monitoring of the skin and education to avoid sun exposure are essential for CLL patients, as data suggest these measures lower NMSC risk. Education and surveillance for SOM are also vital as these significantly shorten survival, and early detection has at least the potential to improve outcomes. Hence adherence to breast and prostate cancer surveillance guidelines, cessation of smoking, evaluation of iron deficiency for potential gastro‐intestinal tract malignancy and investigation of suspicious symptomatology in other organ systems are important. As SPMs occur in over half of all CLL patients, the frequent exclusion of such patients in clinical trials limits our understanding of 'real‐world' outcomes. CLL has seen dramatic improvements in treatment and survival since the introduction of ICT and targeted therapies, and the high incidence and health burden of SPMs may be a key limitation to further progress in this disease.

## CONFLICT OF INTEREST

The authors have no conflict of interest to declare.

## AUTHOR CONTRIBUTIONS

Stephen P. Mulligan, Luke Coyle, Ian Kerridge, William Stevenson, Christopher Arthur, Naomi McKinlay, Keith Fay, Christopher Ward, Matthew Greenwood and Stephen Shumack collected the data. Stephen P. Mulligan and Stephen Shumack confirmed the accuracy of the SC data. Yandong Shen compiled the data for analysis and wrote the first draft of the manuscript. Ann Solterbeck performed the statistical analysis. Yandong Shen and Stephen P. Mulligan interpreted the data and prepared the final manuscript, which all authors reviewed and approved.

## Supporting information

Supporting Information
